# Toward unified molecular surveillance of RSV: A proposal for genotype definition

**DOI:** 10.1111/irv.12715

**Published:** 2020-02-05

**Authors:** Stephanie Goya, Mónica Galiano, Inne Nauwelaers, Alfonsina Trento, Peter J. Openshaw, Alicia S. Mistchenko, Maria Zambon, Mariana Viegas

**Affiliations:** ^1^ Virology Laboratory Ricardo Gutiérrez Children's Hospital Buenos Aires Argentina; ^2^ Consejo Nacional de Investigaciones Científicas y Tecnológicas (CONICET) Buenos Aires Argentina; ^3^ Respiratory Virus Unit National Infection Services Public Health England London UK; ^4^ National Heart and Lung Institute Imperial College London London UK; ^5^ Instituto de Investigación Hospital 12 de Octubre Madrid Spain; ^6^ Comisión de Investigaciones Científicas (CIC) Buenos Aires Argentina; ^7^ Present address: WHO Collaborating Centre for Reference and Research on Influenza Francis Crick Institute London UK

**Keywords:** average genetic distance, genotypes, global molecular surveillance, human orthopneumovirus, human respiratory syncytial virus, lineages, phylogenetic analysis, subgenotypes

## Abstract

**Background:**

Human respiratory syncytial virus (RSV) is classified into antigenic subgroups A and B. Thirteen genotypes have been defined for RSV‐A and 20 for RSV‐B, without any consensus on genotype definition.

**Methods:**

We evaluated clustering of RSV sequences published in GenBank until February 2018 to define genotypes by using maximum likelihood and Bayesian phylogenetic analyses and average p‐distances.

**Results:**

We compared the patterns of sequence clustering of complete genomes; the three surface glycoproteins genes (SH, G, and F, single and concatenated); the ectodomain and the 2nd hypervariable region of G gene. Although complete genome analysis achieved the best resolution, the F, G, and G‐ectodomain phylogenies showed similar topologies with statistical support comparable to complete genome. Based on the widespread geographic representation and large number of available G‐ectodomain sequences, this region was chosen as the minimum region suitable for RSV genotyping. A genotype was defined as a monophyletic cluster of sequences with high statistical support (≥80% bootstrap and ≥0.8 posterior probability), with an intragenotype p‐distance ≤0.03 for both subgroups and an intergenotype p‐distance ≥0.09 for RSV‐A and ≥0.05 for RSV‐B. In this work, the number of genotypes was reduced from 13 to three for RSV‐A (GA1‐GA3) and from 20 to seven for RSV‐B (GB1‐GB7). Within these, two additional levels of classification were defined: subgenotypes and lineages. Signature amino acid substitutions to complement this classification were also identified.

**Conclusions:**

We propose an objective protocol for RSV genotyping suitable for adoption as an international standard to support the global expansion of RSV molecular surveillance.

## 1 BACKGROUND

Human respiratory syncytial virus (RSV) is the commonest viral cause of acute lower respiratory tract infections in children worldwide, being the main infectious reason for pediatric hospitalizations.^1^ There is yet neither an effective vaccine nor antiviral therapy, and treatment remains supportive, although passive antibody prophylaxis (palivizumab) is available in developed countries for prevention of high‐risk babies. Progress is being made with vaccine development with encouraging results reported for live‐attenuated and subunit approaches.^2^

**Figure 1:**
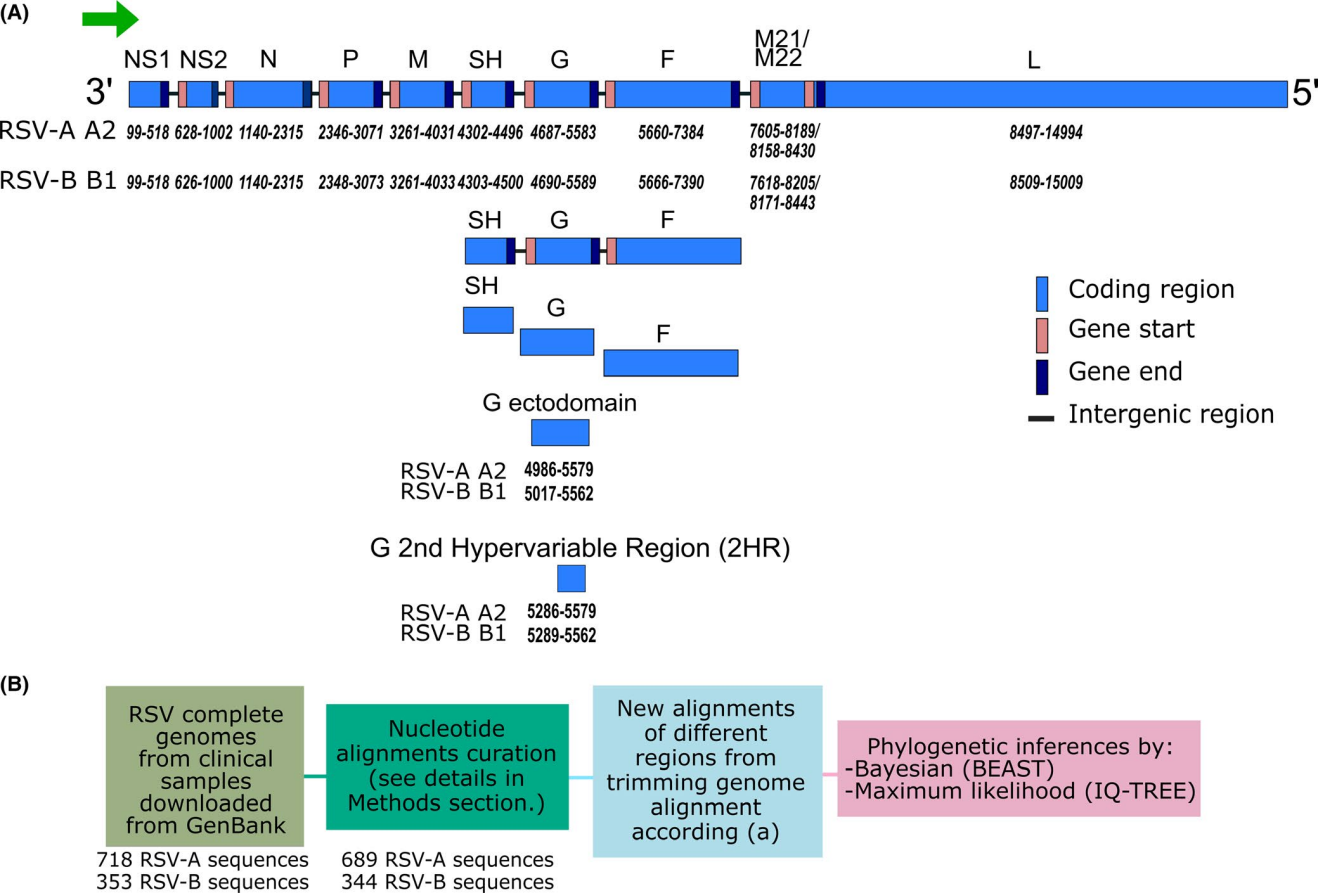
Scheme of RSV genome organization and workflow for different RSV alignments and phylogenies generation. A, RSV genome organization and the regions used for new alignments generated from trimming of the genomes. Numbers below the rectangles show the position of the coding regions in the genome and allocation of G‐ectodomain and G 2nd hypervariable regions, according to the reference strains A2 for RSV‐A (GenBank acc. no.:NC_038235.1) and B1 for RSV‐B (GenBank acc. no.: NC_001781.1). B, Workflow for the phylogenies inference of different regions used for both RSV‐A and B. Numbers of sequences before and after data curation are detailed

RSV is member of the *Orthopneumovirus* genus within the family *Pneumoviridae*.^3^ It is an enveloped virus with a negative‐sense ssRNA genome of ~15 200 nucleotides (nt) in length. Its genome encodes for 11 proteins (Figure 1A). Two antigenic subgroups (A and B) are distinguished by polyclonal and monoclonal antibodies. Both subgroups are evolutionary lineages which diverged approximately 350 years ago^4^ with considerable genotypic variability within them. The major differences are found in the attachment glycoprotein G, which has only 53% amino acid sequence conservation across strains and has been used historically for molecular characterization.^5^

Currently, 13 RSV genotypes have been defined among the subgroup A strains (GA1‐7,^6^, ^7^ SAA1,^8^ NA1‐4,^9^, ^10^ and ON1‐2^11^, ^12^) and 20 genotypes for the subgroup B strains (GB1‐4,^6^ SAB1‐4,^8^ URU1‐2,^13^ and BA1‐10^14^, ^15^) but the criteria used for definition of a genotype varies based on phylogenetic analyses inferred using different methods (maximum likelihood, maximum parsimony, neighbor‐joining or Bayesian inferences). Most definitions focus on clustering of phylogenetic clades with significant bootstrap values (>70%) in trees built from alignments encompassing the 2nd hypervariable region (HR) of G gene (~270 nt in length). To support these definitions, the average genetic distance (p‐distance) has been used, sometimes as an informative tool, sometimes as an arbitrarily selected cut‐off value (<0.07)^8^ or similarity value (>96%).^6^ Overall, there is a lack of consensus regarding the criteria to be used to allocate genotypes. The presence of a duplicated segment (ON −72 nt duplication‐ and BA −60 nt duplication‐ genotypes in RSV‐A and RSV‐B, respectively) in the 2nd HR of G gene has been used as an added criterion to define new genotypes.^11^, ^16^ A more recent proposal for genotyping RSV‐A strains used phylogenetic analysis of the G‐ectodomain and reevaluated historical genotypes using average p‐distances within and among genotypes with a cut‐off value of 0.049, based on the average p‐distance of the oldest RSV‐A genotype, GA1.^17^

Unification of the nomenclature and phylogenetic classification of viruses with high impact in human and animal health, such as highly pathogenic H5N1 avian influenza virus (https://www.who.int/influenza/gisrs_laboratory/h5n1_nomenclature/en/), Newcastle disease virus,^18^ measles virus,^19^ and HCV 20 is an important underpinning principle for unambiguous tracking of virus evolution, which may have significant public health consequences. Reaching consensus on a unified criterion for RSV genotype definition is essential to explore the association of genotype with disease severity, or geographic or temporal restriction of virus circulation. The aim of this work is to reach a new genotype definition based on both phylogenetic analyses and average p‐distances that would bring uniformity to strains designations and thereby facilitate conclusions about viral evolution based on data from surveillance studies.

## 2 METHODS

### 2.1 Sequence datasets

RSV complete genome sequences from human clinical samples were retrieved from GenBank up to February 2018 (718 RSV‐A and 348 RSV‐B sequences). The criteria used to curate the sequences include removing sequences with “NNN” regions, ambiguous nucleotides, and/or sequences with 1‐2 nucleotide deletions causing frameshifts. Sequences with incorrect RSV subgroup allocation were identified and added to the correct subgroup. After curation, a total of 689 complete genomes of RSV‐A and 344 of RSV‐B sequences were obtained (Figure 1B).

Each genomic dataset was aligned with MUSCLE (multiple sequence comparison by log‐expectation).^21^ These were further trimmed to produce alternative datasets encompassing different regions of the RSV genome to be analyzed: the concatenated SH‐G‐F genes (including their intergenic regions) as a single stretch; the three surface glycoprotein genes as separate ORFs, the ectodomain and the 2nd HR of the G gene (Figure 1A).

A second analysis was performed with all the G‐ectodomain sequences published in GenBank up to February 2018 (3362 RSV‐A and 1742 RSV‐B sequences). Alignment curation was performed as described for genome sequences. In addition, identical nucleotide sequences were detected and removed with iq‐tree software and only one representative non‐identical sequence was kept.^22^ After curation, a total of 2481 G‐ectodomain sequences for RSV‐A and 1259 for RSV‐B were obtained.

The final alignments were visually checked for artifacts produced during the alignment procedure.

### 2.2 Phylogenetic inferences

The selection of the most suitable nucleotide substitution models was performed with iq‐tree software according to the Akaike information criterion (AIC).^23^ The GTR + G was the most suitable model for most of the alignments, with exception of complete genome and the three surface glycoprotein alignments in which the GTR + I + G was selected for both subgroups. In addition, TIM + G was the model selected for both SH alignments.

Maximum likelihood trees were inferred using iq‐tree v1.6.7 software with 1000 ultrafast bootstrap replicates plus SH‐like approximate likelihood ratio test as statistical support (values ≥80% were defined as well‐supported).^24^

Bayesian trees were inferred with beast v1.10.2 package.^25^ Demographic and molecular clock model selections were performed by estimating the model marginal log‐likelihood through the path sampling method, and uncorrelated relaxed molecular clock and Skyride tree prior were selected. The number of generations was between 50 and 100 million, and an appropriate sample frequency was used to obtain 10 000 trees. Convergence was assessed by estimating the effective sampling size after a 10% of burn‐in by using tracer v1.7.1 (http://tree.bio.ed.ac.uk/software/tracer/). TreeAnnotator was used to summarize the posterior trees from BEAST into a maximum clade credibility tree (MCCT). The statistical support of the nodes was considered as well‐supported when posterior probabilities were ≥0.8.

A web tool (http://www.phylo.io) was used for comparison of tree topologies.^26^ figtree v1.4.3 software was used for visualization of phylogenies.^27^

Throughout the entire manuscript, we use the term genetic clade to mean a monophyletic cluster with high statistical support (≥80% bootstrap and ≥0.8 posterior probability values) with more than three epidemiologically unrelated sequences, that is, non‐identical sequences from different countries, different outbreaks, or combination of both.

### 2.3 Evaluation of phylogenetic signal

The loss of phylogenetic signal due to substitution saturation was evaluated with dambe software. The level of saturation was studied by plotting the pairwise number of observed transitions and transversions versus genetic distance.^28^

### 2.4 Genetic distances and amino acids analyses

The average genetic distance within and among clades was estimated for alignments with unique sequences with mega7 software with the most simplified method, p‐distance, as a proportion of nucleotide sites at which two sequences being compared were different.^29^ Pairwise deletion was the treatment used for the alignment's gaps. The standard errors of the estimates were determined by the bootstrap method with 1000 replicates.

The treesub program (https://github.com/tamuri/treesub) was used to analyze signature amino acids that support the defined levels of classification. It estimates ML trees using raxml, followed by branch annotation of amino acid substitutions. aliview v1.25 was used to visualize and check signature amino acids.^30^

## 3 RESULTS AND DISCUSSION

For each RSV subgroup, phylogenetic trees by maximum likelihood (ML) and Bayesian inferences were obtained for the alignments of complete genomes, concatenated SH‐G‐F genes (including their intergenic regions), the individual coding regions of the SH, G, and F genes, and the G‐ectodomain and G 2nd hypervariable region (HR). However, convergence was not achieved for Bayesian tree of the RSV‐B SH gene even after 50 million generations. The tree topologies of both ML and Bayesian inferences were similar (ML and Bayesian trees in Figures S1 and S2, respectively). Nevertheless, the longer the length of the region analyzed, the better the resolution of the trees, as seen when full genome sequences are used for the analyses. However, there is uneven representation of full‐length global circulating strains both in number and in countries of origin, when compared to the availability of shorter fragments such as the G‐ectodomain sequences (Figure S3).

The phylogenetic analysis of genomic regions reveals that most of them have high statistical support in the phylogenies' nodes (≥80% bootstrap and ≥0.8 posterior probability values) with exception of the trees obtained with the SH gene and the G 2nd HR. In addition, the plots of the absolute number of transitions and transversions vs divergence reveal loss of phylogenetic signal for both SH gene and the G 2nd HR, while most of the other analyzed regions show a constant increase in the absolute number of transitions and transversions with the increment of divergence (Figure S4).

**Figure 2:**
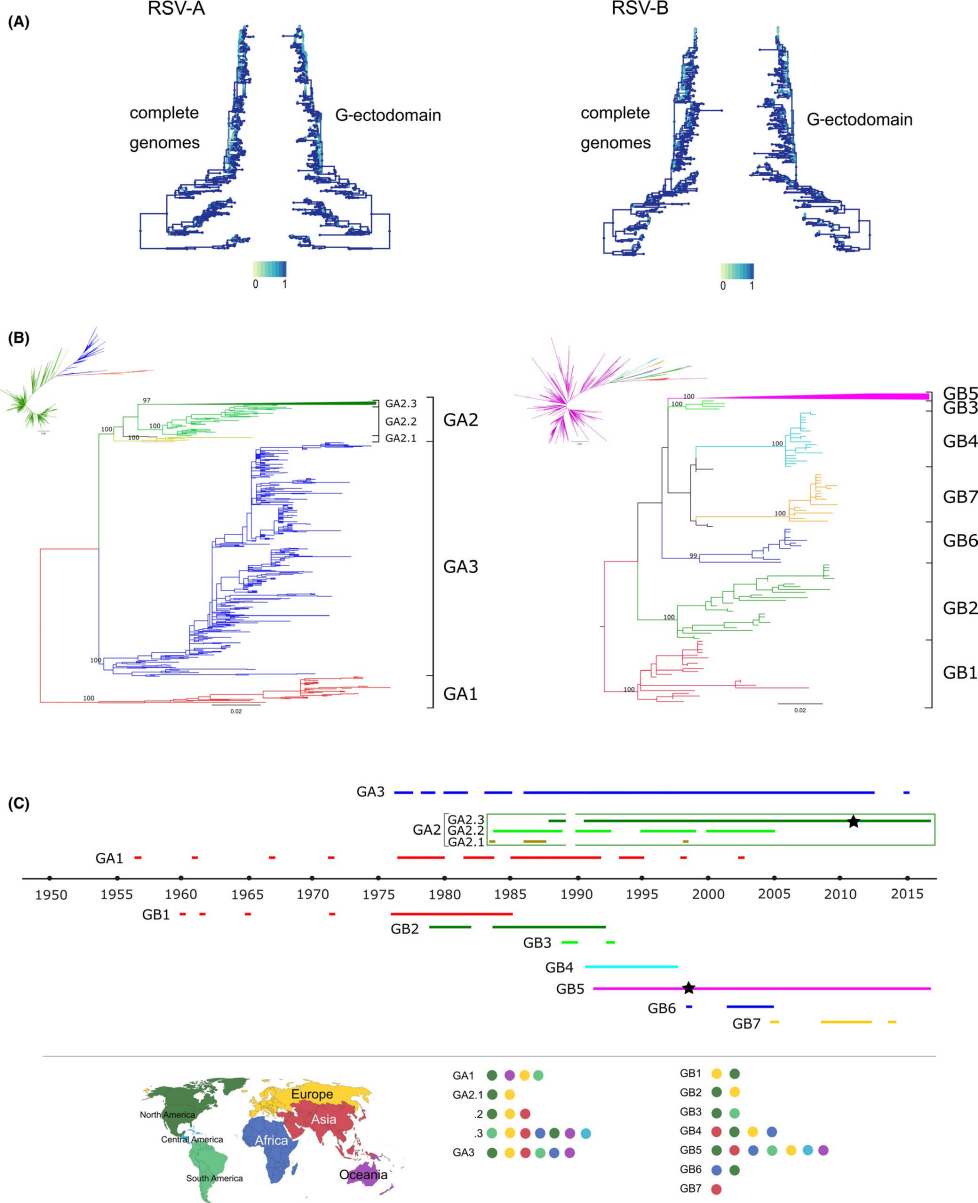
Genotypes definition with G‐ectodomain region. A, Comparison of tree backbones. Trees were constructed from an alignment of complete genome sequences per subgroup. Branches are colored according to each node's similarity to its best corresponding node in the opposite tree. In the scale, 0 means non‐similarity and 1 means exact matching. B, Maximum likelihood trees for RSV‐A and B with G‐ectodomain sequence alignments (see the Section Methods for details). Colors in branches represent genotypes and subgenotypes. Subgenotype GA2.3 and genotype GB5 are collapsed in the rectangular trees. Complete uncollapsed trees are shown in the radial tree small figure next to each rectangular tree. UF bootstrap values of genotypes and subgenotypes nodes are shown. C, Timeline of detection of the identified genotypes and subgenotypes. Colored lines show the years of detection of a given genotype or subgenotype. Stars in lines of GA2 and GB5 genotypes show the year where strains with duplication in the 2nd hypervariable region of G gene were detected (72nt in RSV‐A and 60nt in RSV‐B). Geographical location of genotypes in order of detection is also shown

Phylogenies constructed from F gene, G gene, and G‐ectodomain showed very similar topologies, albeit with lower resolution than complete genome trees. Given that vaccine and prophylaxis strategies are mostly targeted to the F protein, analysis of F sequences would provide useful data; however, there is more availability both in number and geographical representation of G‐ectodomain sequences. Therefore, we focused on G‐ectodomain. A comparison of the tree topology of the complete genome and the G‐ectodomain phylogenies is shown in Figure 2A. Even though several clusters are not identical between complete genomes and G‐ectodomain phylogenies, the detailed analysis by taxa shows that the inconsistencies occur largely in terminal nodes with low statistical support. The ancestor nodes of the main clades of sequences remain unchanged in their topology and statistical support, suggesting that potential genotype assignation of the sequences would not be influenced.

Based on these results, we conclude that the G‐ectodomain should be considered the minimum region of the genome to be analyzed to obtain reliable phylogenies and genotype designation, and it can be used to obtain robust phylogeographic analyses allowing inferences about the origin of viral introductions in a particular geographical region.

Next, to classify genotypes, we based on the strategy of using average p‐distances for RSV‐A subgroup, described in 2015.^17^ Briefly, in that work the average p‐distances among individual genotypes, as well as within each genotype, were calculated. The highest intragenotype average p‐distance was found for the GA1 genotype. This value was taken as the threshold for sorting viruses into different genotypes. Using these criteria, we reevaluated and redefined all previous genotypes as explained below and also summarized in Table S1.

One alignment of each RSV subgroup was obtained from all available curated G‐ectodomain sequences up to February 2018. Phylogenetic analyses by two inference methods (ML and Bayesian) were performed. Monophyletic clades with high statistical support (≥80% for bootstrap and ≥0.8 for posterior probabilities values) that clustered the oldest or first detected strains were identified and designated as GA1 for RSV‐A and GB1 for RSV‐B. A total of 44 non‐identical G‐ectodomain sequences (64 total sequences) were associated with GA1 and 22 non‐identical G‐ectodomain sequences (30 total sequences) with GB1. The average intragenotype p‐distance for GA1 was 0.036 (SE:0.004), and for GB1 was 0.032 (SE:0.004); thus, an intragenotype p‐distance of 0.03 (3% divergence) was set as a general cut‐off value for both subgroups. Based on this defined cut‐off value, the smallest number of well‐supported genetic clades was identified and the average intraclade p‐distance was calculated, and when the value resulted equal or below 0.03, the clade was defined as genotype (Tables 1A and 2). As a result, three genotypes were identified for RSV‐A (GA1‐GA3) and seven genotypes for RSV‐B (GB1‐GB7). This collapses the previously larger number of groupings into a smaller set of genotypes for both RSV‐A and RSV‐B.

**Table 1:** Estimates of average genetic distances among and within RSV‐A genotypes (A) and subgenotypes (B)

(A)			
	GA1 (44)	GA2 (2050)	GA3 (387)
GA1 (44)	**0.036 SE:0.004**		
GA2 (2050)	0.13 SE:0.01	**0.032 SE:0.003**	
GA3 (387)	0.13 SE:0.01	0.096 SE:0.009	**0.035 SE:0.003**

(B)			
	GA2.1 (8)	GA2.2 (52)	GA2.3 (1988)
GA2.1 (8)	**0.020 SE:0.003**		
GA2.2 (52)	0.058 SE:0.008	**0.028 SE:0.003**	
GA2.3 (1988)	0.074 SE:0.009	0.073 SE:0.009	**0.030 SE:0.003**

Note

Calculations were done for genotypes defined from a phylogenetic tree with 2481 unique RSV‐A sequences of the G‐ectodomain region. The number of sequences of each genotype (A) or subgenotype (B) is shown in parenthesis. P‐distances were calculated between and among individual genotypes (A) or subgenotypes (B). P‐distances within each genotype and subgenotype are denoted in bold. Standard error (SE) estimates are shown next to the average genetic distances.

**Table 2:** Estimates of average genetic distances among and within RSV‐B genotypes

	GB1 (22)	GB2 (28)	GB3 (4)	GB4 (21)	GB5 (1149)	GB6 (13)	GB7 (18)
GB1 (22)	**0.032 SE:0.004**						
GB2 (28)	0.072 SE:0.008	**0.037 SE:0.004**					
GB3 (4)	0.073 SE:0.008	0.064 SE:0.007	**0.015 SE:0.002**				
GB4 (21)	0.08 SE:0.01	0.077 SE:0.009	0.066 SE:0.009	**0.011 SE:0.002**			
GB5 (1149)	0.087 SE:0.008	0.077 SE:0.007	0.072 SE:0.008	0.083 SE:0.008	**0.032 SE:0.003**		
GB6 (13)	0.059 SE:0.007	0.056 SE:0.006	0.049 SE:0.007	0.059 SE:0.008	0.061 SE:0.008	**0.017 SE:0.004**	
GB7 (18)	0.074 SE:0.009	0.068 SE:0.008	0.064 SE:0.009	0.066 SE:0.009	0.075 SE:0.009	0.052 SE:0.007	**0.022 SE:0.003**

Note

Calculations were done for genotypes defined from a phylogenetic tree with 1259 unique RSV‐B sequences of the G‐ectodomain region. The number of sequences of each genotype is shown in parenthesis. P‐distances within genotypes are denoted in bold. Standard error (SE) estimates are shown next to the average genetic distances.

For a standardized genotype denomination, we propose to adopt the nomenclature defined by Peret et al (1998) where genotypes are designated as GAX and GBX; G stands for G‐based genotype, A or B designate RSV subgroups, and X corresponds to a first‐order ascending numbering system. This is a straightforward definition which does not allude to geographic references, avoiding potential stigmatizing labeling of RSV clades.^6^ We renamed all genotypes in ascending order according to their first detection date, except GA2. We maintained its designation despite having emerged more recently than GA3, because it is a current widespread‐circulating genotype and renaming this genotype could create confusion in the RSV community.

The minimum average intergenotype p‐distance was 0.097 SE:0.008 (9% divergence) for RSV‐A genotypes and 0.056 SE:0.006 (5% divergence) for RSV‐B (Tables 1A and 2).

Small numbers of independent sequences not fitting the genotype definition were not classified and will remain undefined until future sequences cluster with them and meet the definition of genotype. It is also possible that these sequences represent extinct viral genotypes.

Genotypes GA2, GA3, and GB5 are currently the most frequently detected and the largest ones (Figure 2B,C). Within these genotypes, further levels of classification were defined.

The next level of classification, defined as subgenotypes, encompassed well‐supported dichotomies (≥80% bootstrap and ≥0.8 posterior probability values) with an average intersubgenotype p‐distance smaller than the minimum intergenotype p‐distance described (divergence: 9% for RSV‐A and 5% for RSV‐B). The subgenotype nomenclature was defined as GAX.Y or GBX.Y, where X is the genotype and Y the subgenotype and corresponds to a second‐order ascending numbering system. Within GA2, three subgenotypes were identified (GA2.1‐GA2.3; Figure 2B) with average p‐distances among them as shown in Table 1B. GA3 and GB5 did not show well‐supported dichotomies within them. However, we cannot rule out the possibility that an increasing number of available sequences in the future will allow improved resolution of subgenotypes. For this reason, we propose to designate the level related to subgenotypes with a 0 (zero) until further resolution is possible.

Finally, in order to facilitate global surveillance, a further level of classification within subgenotypes was defined as lineages. To find an appropriate definition, we evaluated an alternative strategy to deal with the presence of polytomies in the trees, where lineages were defined according to the divergence of the strains from the ancestral node of the subgenotype. The allocation of lineages within each subgenotype was defined by the estimation of patristic distances (set as the sum of the lengths of the branches that link two nodes in a tree) between the internal nodes and the ancestral node of the subgenotype.

**Figure 3:**
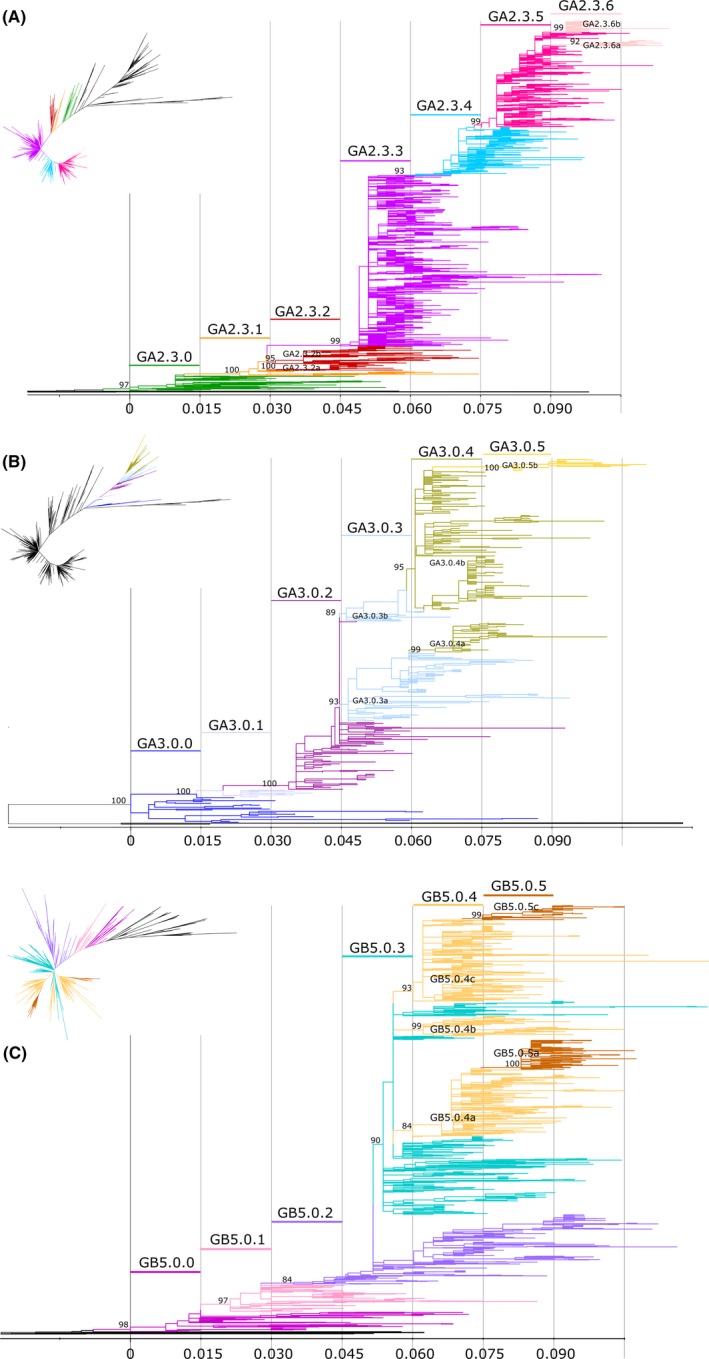
Lineages identified in the subgenotype GA2.3, GA3.0 and GB5.0. Maximum likelihood trees in rectangular format showing lineages in subgenotypes: GA2.3 (A), GA3.0 (B) and GB5.0 (C). Colors in branches denote different lineages. The name of each lineage is shown above the colored line according to that lineage. Scale bar shows patristic distance by ranges of 0.015. UF Bootstrap of each ancestral lineage node is detailed. The small figure of the radial tree shows colored lineages in the uncollapsed whole trees for RSV‐A or B, only the subgenotype lineages are denoted, the rest of the tree is colored in black

From the ML tree rooted with GA1 and GB1 genotypes, we visualized the nodes/taxas in increasing order of divergence. We assigned a patristic distance of 0 (zero) to the ancestral node of each subgenotype and set a cut‐off of 0.015 patristic distance to define lineages (Figure 3). As we moved across the tree toward the terminal branches, when the node of a well‐supported genetic clade showed a patristic distance ≥0.015 from the subgenotype ancestral node, it was defined as a new lineage. Subsequent lineages were therefore defined when diverged from the subgenotype ancestral node in multiples of 0.015 of patristic distance (>0.030, >0.045 and so on). The lineage nomenclature was defined as GAX.Y.Z or GBX.Y.Z where X is the genotype, Y the subgenotype, and Z corresponds to a third‐order ascending numbering system.

As shown in Figure 3, six lineages were identified within GA2.3 subgenotype (GA2.3.1‐GA2.3.6), five within GA3.0 (GA3.0.1‐GA3.0.5), and five within GB5.0 (GB5.0.1‐GB5.0.5).

When two or more divergent genetic clades, each having a distinct ancestral node, met the definition for new lineages within the same range of patristic distance, a lowercase letter after the lineage number was used to distinguish them. This was the case for GA3.0.3a and GA3.0.3b lineages, and GB5.0.4a, GB5.0.4b, and GB5.0.4c lineages. Once two or more particular lineages were defined with a letter, subsequent lineages were also defined with the same letter to denote the same ancestral origin, such as GB5.0.4a and GB5.0.5a (Figure 3C).

To complement the defined classification levels by phylogenetic clustering, we explored the search of signature amino acids, as used for influenza.^31^ Signature amino acids are ideally defined by substitutions that are uniquely present in all sequences of a given clade. Table 3 and Figures S5 and S6 show amino acid substitutions across genotypes, subgenotypes, and lineages. Although most RSV‐A and B genotypes, subgenotypes and lineages showed substitutions fitting this definition (defined as “main” in Table 3), there were other signature amino acids present in most but not all sequences within a clade, or present in more than one clade across the trees (defined as “secondary” in Table 3). Our analysis shows that all genotypes and subgenotypes in RSV‐A and B were clearly defined by a collection of characteristic amino acid substitutions. For some lineages, there were no unique signature substitutions or at least two concomitant substitutions need to be present for a sequence to be properly classified into that lineage. Thus, we recommend that signature amino acids should be analyzed as a collection of substitutions (or haplotypes) defining each genotype/subgenotype/lineage and used as a combined tool together with phylogenetic methods to further confirm the classification of sequences into given genetic clades.

**Table 3:** Signature amino acids characterizing genotypes/subgenotypes/lineages for RSV‐A (A) and B (B)

(A)	
RSV‐A genotypes	Signature amino acids
GA1	**101P,104P,111I,121G,**123K,141A,***157*S,244I/T,258L,262M,280S,293P**
GA2	**I111P/S/F,** *P226L/H*
GA2.1	**K229N/S**
GA2.2	**V122A,[*I114T* + *L115P*+*S117P/L*]**
GA2.3	**V122A,** *P156Q,P289S/Y/F,S269T*
GA2.3.1	*as GA2.3 plus T252I,G254E*
GA2.3.2	as GA2.3 plus *T133I*
GA2.3.2a	as GA2.3.2 plus *N242S,V225I*
GA2.3.2b	as GA2.3.2 plus **T112I,I141M,R244K/N**
GA2.3.3	as GA2.3 plus *P111S,* **P292S/L**
GA2.3.4	as GA2.3 plus *F208L,* **N273Y/H**
GA2.3.5	as GA2.3 plus *insertion 261‐284* **,E232G/R,T253K/R**
GA2.3.6a	as GA2.3.5 plus **[*V225A + L274P*** * **]**
GA2.3.6b	as GA2.3.5 plus **[P206Q + L274P/S** * **]**
GA3	*I111T,* **S250N**
GA3.0.0	*I111T,* **S250N**
GA3.0.1	**S102F,N191S,** *T295I*
GA3.0.2	as above plus *T113A,T125I,* **P274T**
GA3.0.3	as above plus *N161D,N297D*
GA3.0.3a	as GA3.0.3 plus **E232D**
GA3.0.4a	as GA3.0.3a plus **T227S**
GA3.0.3b	as GA3.0.3
GA3.0.4b	as GA3.0.3b plus *S217P,S224P,P246L,P206Q*
GA3.0.5b	as GA3.0.4b plus **N153K.**

(B)	
RSV‐B genotypes	Signature amino acids
GB1	*137I,150S,152* **S** *,208I,222* **M** *,271S,280I*
GB2	*I118T,* **P223T** *,S257L*
GB3	**R136I**
GB4	*P216S,* **P219S,M222A,** *K224R,* **N230D,K234E,Q248R,K258D**
GB5	**T143N,L237P/S**
GB5.0.0	**T143N,L237P/S**
GB5.0.1	as above, plus insertion *245‐264,S247P* *
GB5.0.2	as above, plus *T138S/F* **,** *K158‐P159del* a *,L223P*
GB5.0.3	as above, plus *V271A*
GB5.0.4a	as GB5.0.3 plus **[** *I200T + I281T* **]**
GB5.0.5a	as GB5.0.4a plus **R136T,T254I** *
GB5.0.4b	as GB5.0.3 plus **[** *S267L + S297F/L* **]** and **K153R** or *I111T*
GB5.0.4c	as GB5.0.3 plus **S291L** or **E305D**
GB5.0.5c	as GB5.0.4c subclade **S291L**,plus *R262G* *
GB6	*A116T,* **T126K,** *P235L,* **Q262L/P**
GB7	*Q180R* **,R214I**,*T239I,* **R242K,V251M/I,** *E272D*

Note

Amino acid annotation follows the format “X122Y,” where X = reference, Y = signature, number = position from beginning of G‐ORF. Signature amino acids are defined against the consensus sequence of GA1 for RSV‐A and GB1 for RSV‐B. “Main” amino acid signatures are in bold, “secondary” changes are in italics. Substitutions in square brackets are needed in combination to be considered as signature amino acids.

a alternative position for this deletion: P159‐K160del.

* Substitutions located within the duplication region of the G gene (72nt in RSV‐A or 60nt in RSV‐B). For these substitutions, the positions were described using strains JN257693_CAN_2010 (RSV‐A) and DQ227364_ARG_1999 (RSV‐B) as reference sequences.

With this new classification, the number of genotypes was reduced from 13 to three genotypes for RSV‐A (GA1‐GA3) and from 20 to seven genotypes for RSV‐B (GB1‐GB7). Two further levels of classification were added: subgenotypes and lineages (summarized definitions in Table S2). The timeline of detection of genotypes and subgenotypes is shown in Figure 2C. In addition, the information about period and regions of circulation, and the first detected strain for all the proposed taxonomic groups is listed in Table 4. For RSV‐A, GA1 is the oldest genotype and was no longer detected after 2003. GA2 and GA3 genotypes currently circulate worldwide, but GA2 is the main circulating genotype and includes the strains with 72nt duplication in the 2nd HR of G (formerly named as ON1 strains) first described in 2011.^11^ In the case of RSV‐B, GB1 was first detected in 1960 and ceased its detection in 1985. GB5 and GB7 are the currently circulating genotypes, whereas GB6 was last detected in 2005. Only sequences from Thailand, Taiwan, China, and India clustered in GB7, showing geographical restriction within the Asian continent, unlike GB5, which shows a global circulation. Most GB5 sequences have the 60nt duplication in the 2nd HR of G, first described in 1999.^15^ The schematic diagram of genotypes and subgenotypes circulation (Figure 2C) and the information listed in Table 4 are a snapshot and may not represent the real global circulation pattern of RSV due to bias in GenBank deposition practices.

**Table 4:** Information about period and regions of circulation, and the first detected strain for all the proposed taxonomic groups

Subgroup	Genotype	Subgenotype	Lineage	Period detected^a^	Regions of circulation^b^	First detected strain
A	1			1956‐2003	North America, South America, Europe, Oceania	AY911262
	2	2.1		1984‐1998	North America, Europe	KP258733
		2.2		1984‐2005	Asia, North America, Europe	HQ731720
		2.3	2.3.0	1977‐2012	Asia, Africa, North America, South America, Europe	KU316166
			2.3.1	1997‐2009	Asia, Africa, North America, South America, Europe, Oceania	AF193324
			2.3.2a	1999‐2006	Africa, South America, Europe	AY343612
			2.3.2b	1999‐2008	Asia, Africa, North America, South America, Europe	AY343603
			2.3.3	2004‐2015	Global	MF496446
			2.3.4	2003‐2016	Global	JX513373
			2.3.5	2010‐2016	Global	JN257693
			2.3.6a	2011‐2015	South America, Europe	JX513365
			2.3.6b	2013‐2015	Africa	KX453341
	3	3.0	3.0.0	1976‐1997	Asia, North America, Europe	HQ731715
			3.0.1	1982‐1990	North America, Europe	MG642031
			3.0.2	1989‐2003	Asia, North America, South America, Europe	AY343587
			3.0.3a	1998‐2011	Asia, North America, South America, Europe, Oceania	JX069802
			3.0.3b	2000‐2006	North America, South America, Europe	AY343561
			3.0.4a	1999‐2009	South America, Europe	EU025205
			3.0.4b	2000‐2013	Asia, Africa, North America, South America, Europe, Oceania	AY343558
			3.0.5b	2006‐2015	Asia, Africa, North America, South America, Europe, Oceania	JX645865
B	1			1960‐1985	North America, Europe	M73545
	2			1979‐1993	North America, Europe	KP258712
	3			1989‐1993	North America, South America	M73543
	4			1991‐1997	Asia, Africa, North America, Europe	AF193332
	5	5.0	5.0.0	1992‐2012	Asia, Africa, North America, South America, Europe	KP258745
			5.0.1	1999‐2012	Asia, Africa, North America, South America, Europe	AY333364
			5.0.2	2004‐2015	Global	JX489436
			5.0.3	2005‐2014	Global	JX576756
			5.0.4a	2008‐2015	Asia, Africa, North America, South America, Europe, Oceania	MF496630
			5.0.4b	2004‐2011	Asia, Africa, America, Europe	DQ227395
			5.0.4c	2007‐2015	Global	KC297480
			5.0.5a	2013‐2016	Asia, Africa, North America, South America, Europe, Oceania	KY249660
			5.0.5c	2010‐2016	Asia, Africa	KF246600
	6			1998‐2005	Africa, North America	JF704213
	7			2005‐2014	Asia	MF496621

a Period detected up to February 2018.

b This may not represent the current real global circulation pattern of RSV due to bias in GenBank deposition practices.

For epidemiological purposes, the proposed procedure to classify newly sequenced strains into existing genotypes/subgenotypes/lineages is listed in Table S3. To facilitate the classification of any new sequence, curated reference alignments for both subgroups A and B are offered as supplementary materials (RSV‐A‐GectodomainReferenceAlignment and RSV‐B‐GectodomainReferenceAlignment). Figures S5 and S6 show ML trees obtained with these alignments (Bayesian inferences upon request). Both trees show congruence with ML trees inferred using the larger datasets for A and B depicted in Figures 2B and 3; therefore, they can be used for rapid classification of new sequences.

The instructions on how to define a new genotype/subgenotype or a lineage are summarized in Table S4 and are based on the procedures described above. As a caveat, the definition of new genotypes, subgenotypes, or lineages should be performed by using all the available G‐ectodomain nucleotide sequences, because when sub‐sampling datasets are obtained, the diversity of the lineages could be underrepresented. We propose that a global consortium of experts in RSV evolution working together with surveillance reference laboratories should be set up to assess the emergence of new genotypes, subgenotypes or lineages, or reassess existing nomenclature or definition on a regular basis. This consortium could form part of the International RSV Society which is a special interest group of the International Society for Influenza and other Respiratory Virus Diseases (isirv; https://www.isirv.org/site/index.php/special-interest-groups/international-respiratory-syncytial-virus-society ). This consortium would be an ideal group to maintain updated reference datasets using curated reference sequences representing each of the defined classification levels, to be used by the global community.

### 3.1 Strengths and weaknesses of this RSV strain classification proposal

All of the analyses in the present study were carried out with sequences downloaded from GenBank, which is not a dedicated and curated database. A variety of issues with the sequences were found that might have affected tree topologies and calculation of average p‐distances. Therefore, a reliable well‐maintained database with curated sequences will be essential to standardize RSV molecular surveillance.

Our study shows that full genome sequences are the most informative and desirable dataset for genotyping purposes. As technology progresses and NGS methodologies become widespread in the near future, costs will eventually reduce enabling more laboratories to implement protocols to generate full viral genomes for their molecular analyses. The strength of this proposal is that genotype, subgenotype, and lineage definitions proposed here are scalable to the complete genome. The cut‐off value for the average p‐distance should be recalculated as shown in Table S5, and patristic distances should be evaluated for the identification of lineages. The strategy proposed in this work relies on the current availability of partial RSV sequences. The results of this study and other publications support the idea that the G‐ectodomain sequences can be effectively used for genetic characterization of RSV strains.^32^, ^33^, ^34^

It is important to highlight the economic benefit of a low‐cost methodology especially for low‐ and middle‐income countries that may be expanding their sequencing activity, given that the length of G‐ectodomain region can be sequenced by Sanger methodology with only two reactions, allowing surveillance laboratories to monitor molecular diversity easily and in real time. In this effort to standardize the molecular classification of RSV strains, we consider that the use of a cut‐off value for genotype definition is essential.

Defining the average intragenotype p‐distance of GA1 and GB1 as cut‐off is very useful because these clades include the oldest strains detected for each subgroup and they also are no longer detected in the population. Although unlikely, we cannot rule out the possibility that in the near future, strains from these two genotypes might still be detected. Genetic distance measure is sensitive to diversity of sampled sequences; thus, if more sequences from old strains were to be released in the future, this could alter average p‐distances of intra/interclades and even slightly modify the cut‐off value. We strongly reinforce the need for periodical reevaluation of genotype definitions by a global consortium of RSV experts.

## 4 CONCLUSION

With many new vaccine candidates in prospect, widespread adoption of a unified criterion for RSV genotyping will enable standardized, comparable analyses across the scientific research and public health communities. Thus, our proposal of RSV strain classification will facilitate the analysis of strains in clinical and epidemiological studies and would be a fitting start to a joint global RSV surveillance.

## CONFLICT OF INTEREST

The authors declare that they have no competing interests.

## DEDICATION

We would like to dedicate this manuscript to the memory of Dr José Antonio Melero, who was an outstanding researcher and warm and generous individual who encouraged us to work on this topic, with whom we began to work on the unification of the RSV genotype definition shortly before his untimely death.
