# Supplementary figures and images for: Toward unified molecular surveillance of RSV: A proposal for genotype definition
Source: Influenza Other Respir Viruses. 2020 Feb 5;14(3):274–85. doi: 10.1111/irv.12715 (PMC7182609; doi:10.1111/irv.12715)

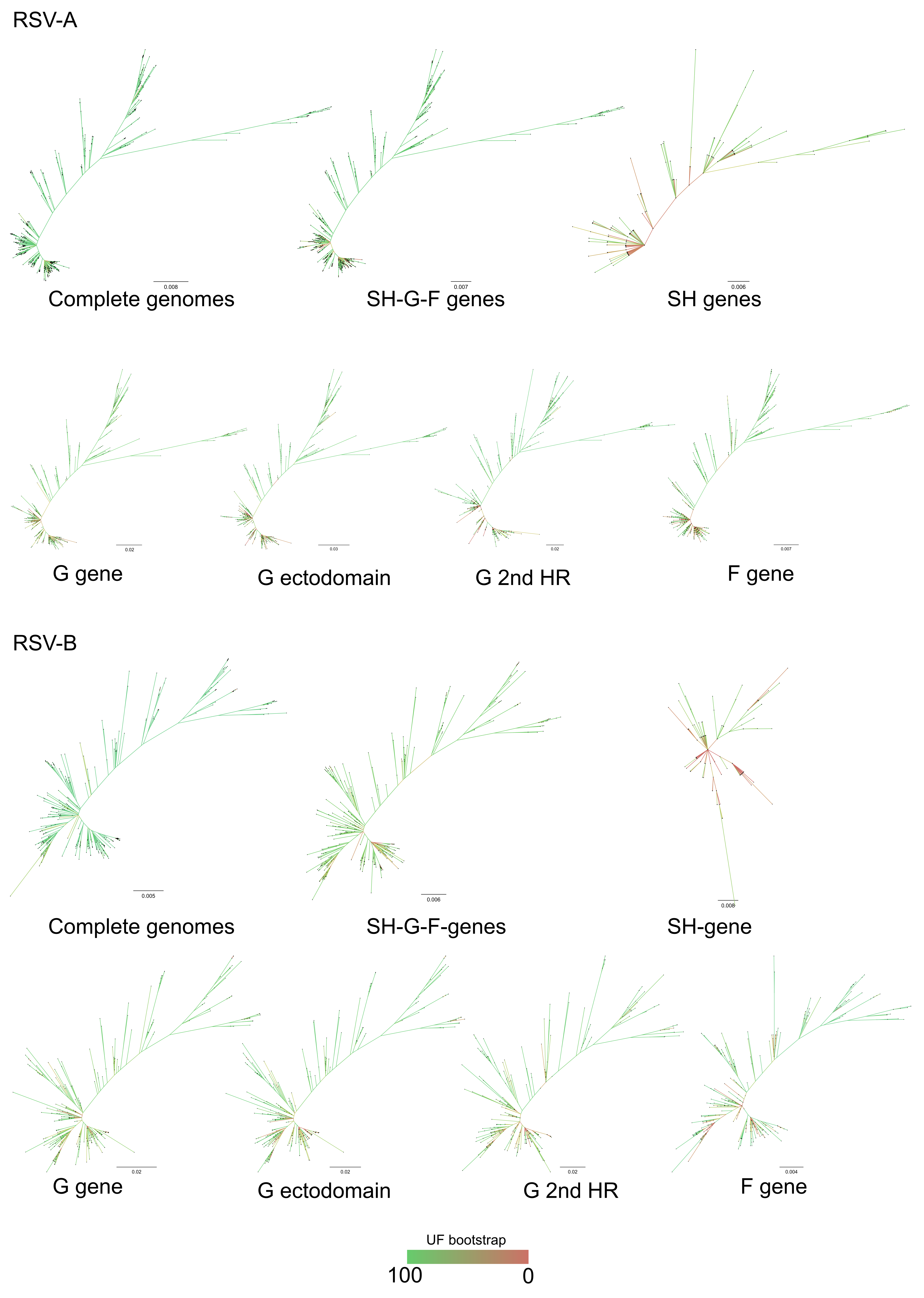

Supplement: Supplementary file 1 [file IRV-14-274-s001.png]

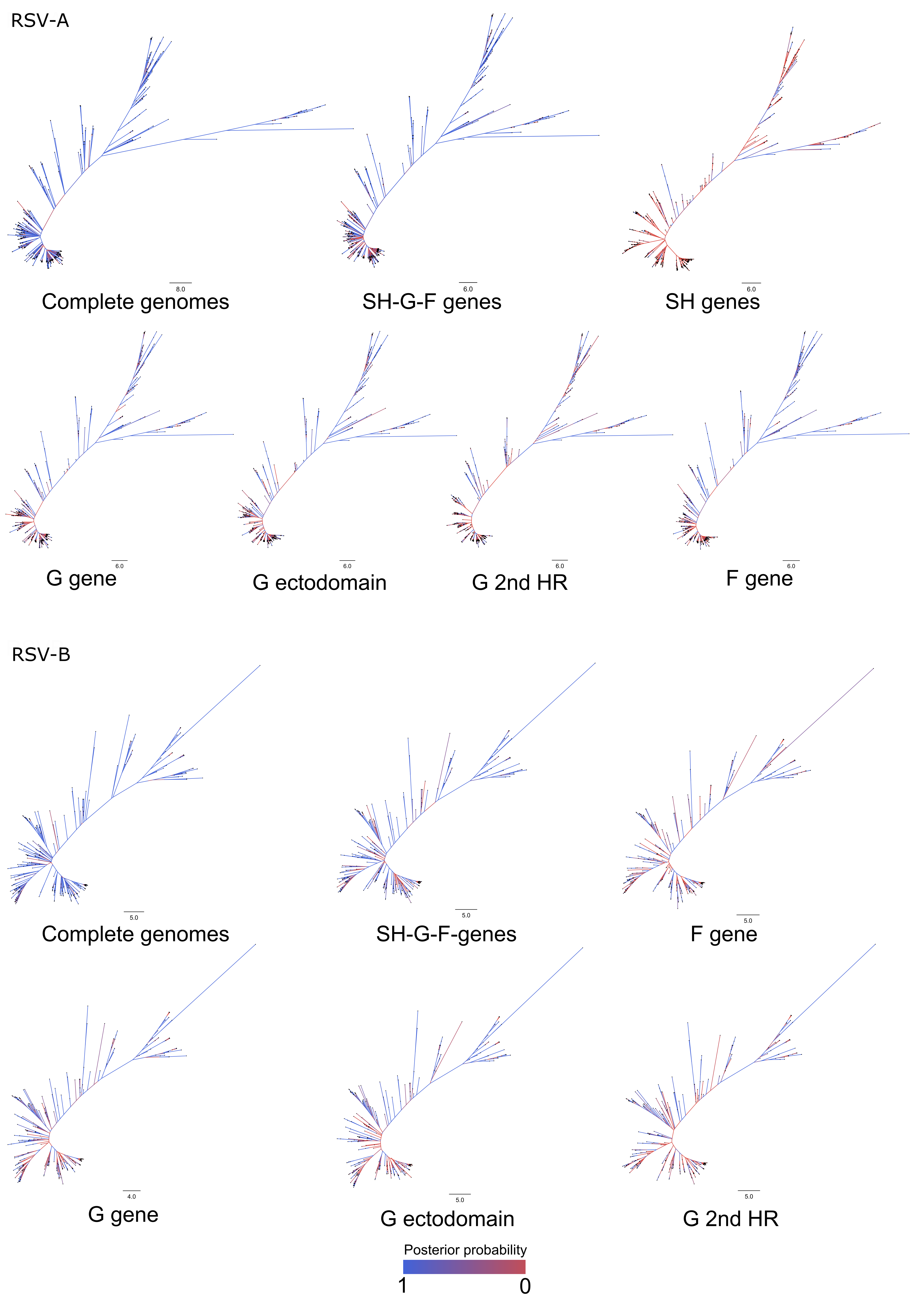

Supplement: Supplementary file 2 [file IRV-14-274-s002.png]

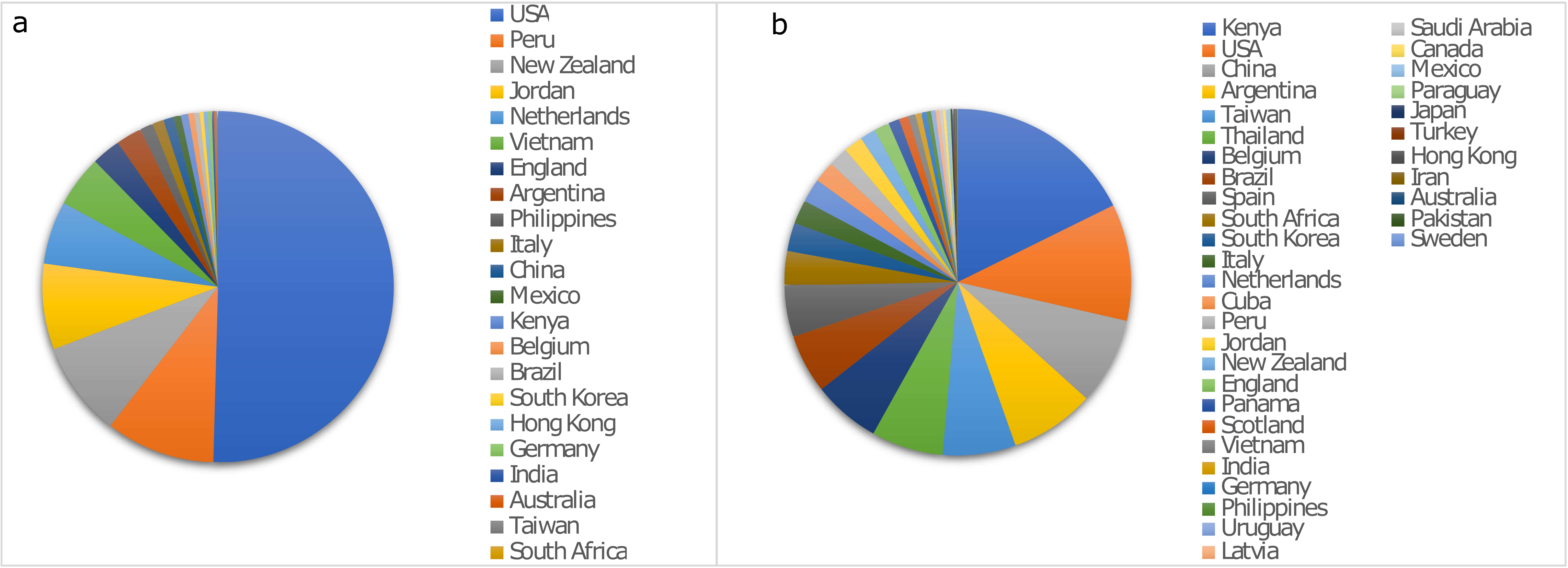

Supplement: Supplementary file 3 [file IRV-14-274-s003.png]

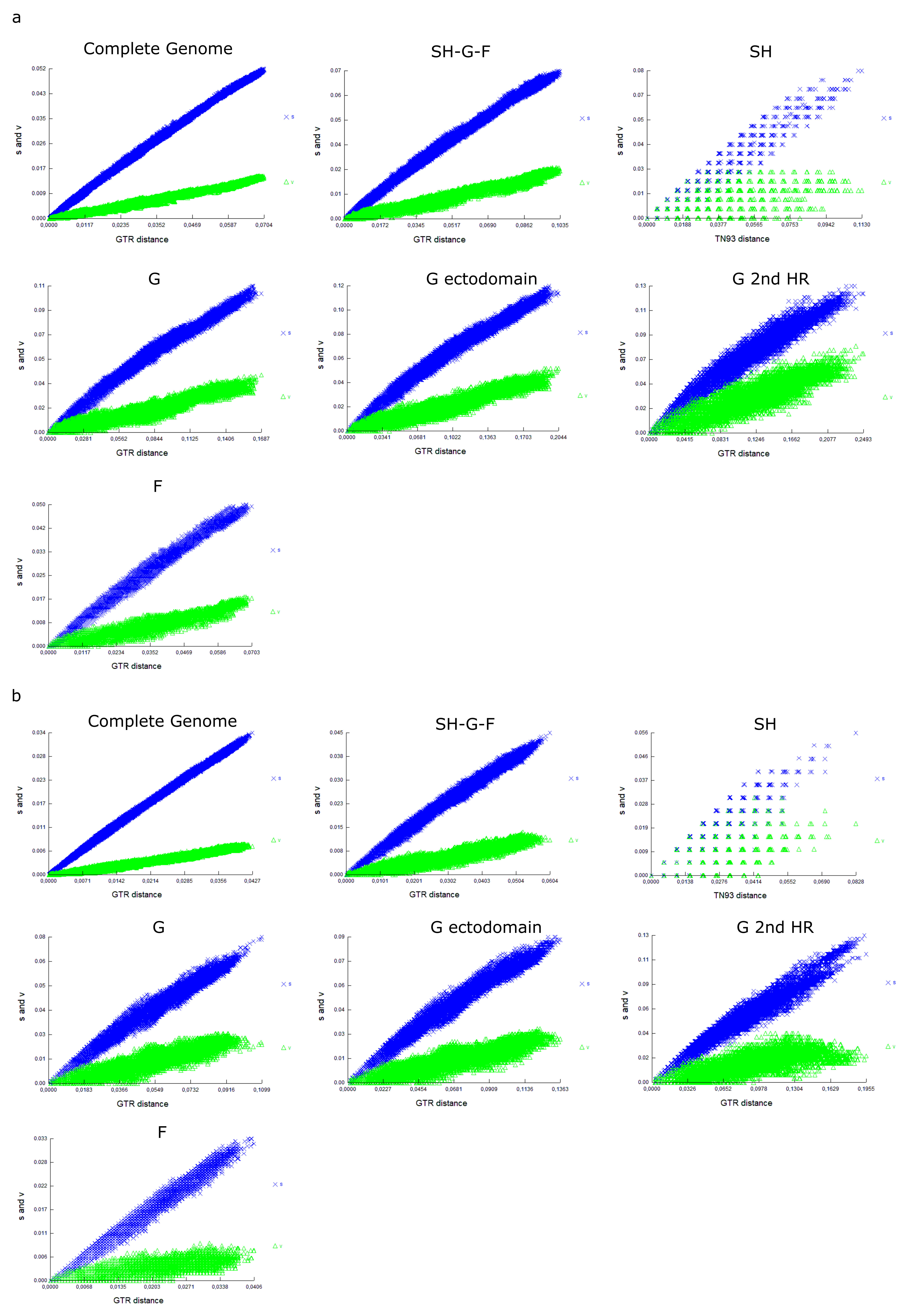

Supplement: Supplementary file 4 [file IRV-14-274-s004.png]

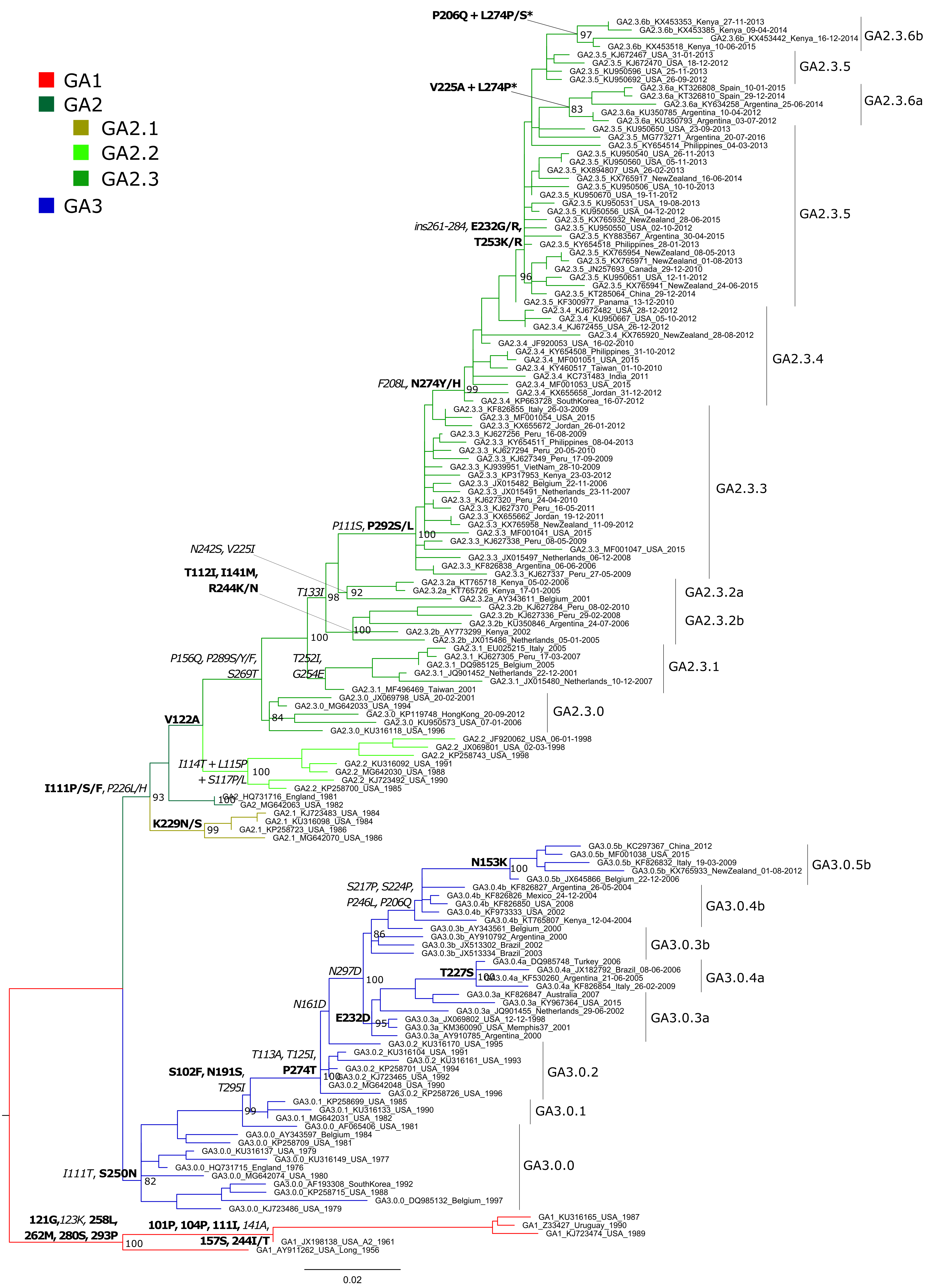

Supplement: Supplementary file 5 [file IRV-14-274-s005.png]

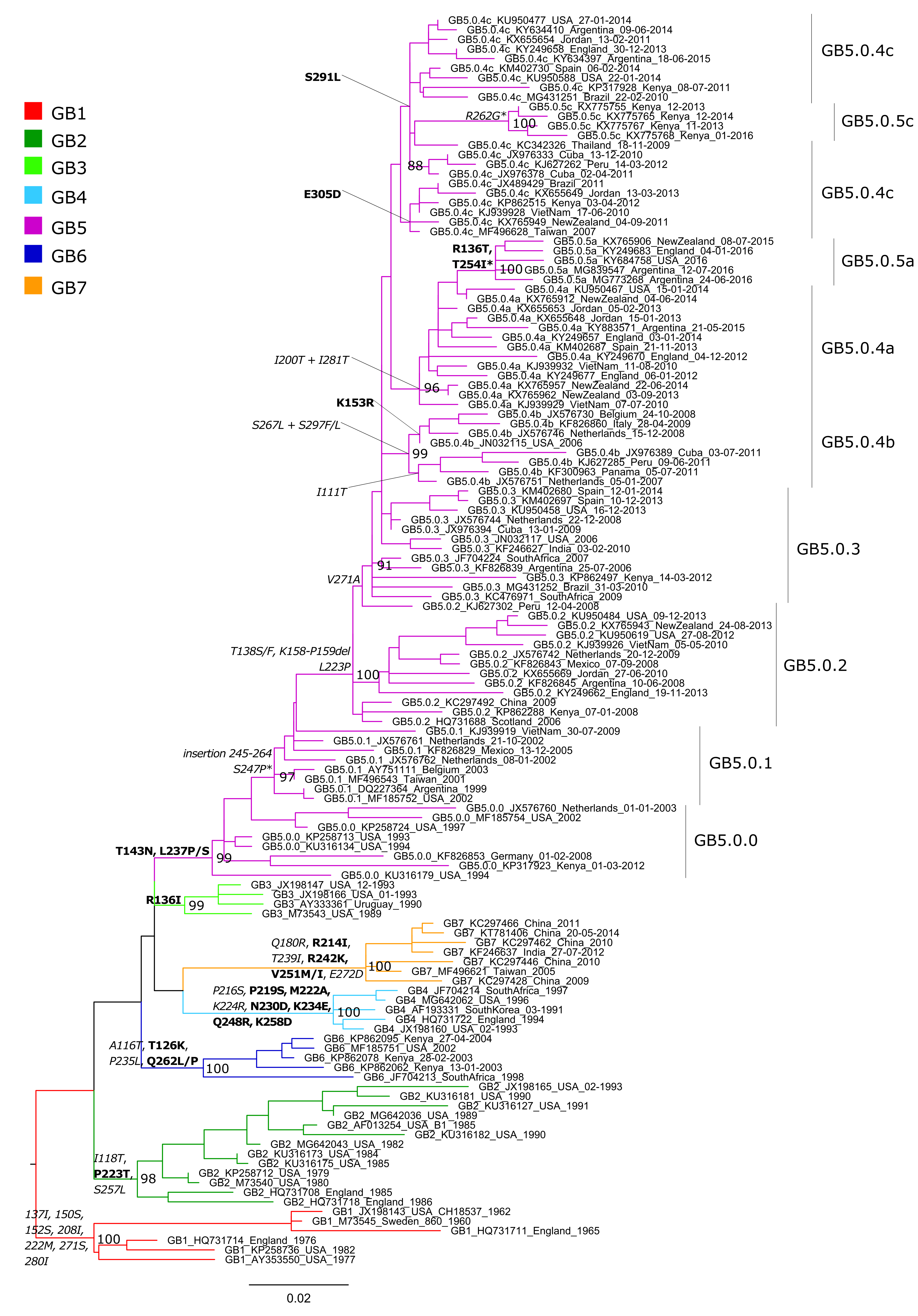

Supplement: Supplementary file 6 [file IRV-14-274-s006.png]
