# Supplementary material for: Toward unified molecular surveillance of RSV: A proposal for genotype definition
Source: Influenza Other Respir Viruses. 2020 Feb 5;14(3):274–85. doi: 10.1111/irv.12715 (PMC7182609; doi:10.1111/irv.12715)
Supplement: Supplementary file 7 [file IRV-14-274-s007.docx]

**Supplementary table 1. Procedure followed for genotypes definition.**

| **1** | One alignment of each RSV subgroup was obtained from all available G-ectodomain sequences downloaded from GenBank up to February 2018 and curated as described in Material and Methods. |
| --- | --- |
| **2** | Phylogenetic analyses by two inference methods (Bayesian and Maximum Likelihood) were performed with each alignment. |
| **3** | Monophyletic clade with high statistical support (≥80% for bootstrap for ML and ≥0.8 for posterior probability for Bayesian analysis) that clustered the oldest strains detected was identified. The designation of GA1 genotype and GB1 genotype for RSV-A and B, respectively was allocated to that clade. |
| **4** | The average intragenotype genetic distance (p-distance) for GA1 and GB1 was calculated. The p-distance values were 0.036 (SE:0.004) for RSV-A, and 0.032 (SE:0.004) for RSV-B, thus an average intragenotype p-distance of 0.03 or a divergence of 3% was set as a general cut-off value for both subgroups. |
| **5** | The phylogenetic trees for each subgroup were rooted with GA1 and GB1 for RSV-A and B, respectively. |
| **6** | The minor number of genetic clades with high statistical support (≥80% for bootstrap for ML and ≥0.8 for posterior probability for Bayesian analysis) and with an average intraclade p-distance ≤0.03 were identified and named according genotype nomenclature. |
| **7** | If an identified independent genetic clade had a greater average intraclade p-distance than the cut-off value, it was divided into the smallest number of clades that met the definition of genotype proposed in the above points. |

**Supplementary table 2. Definitions of different levels of classification.**

| **Genotype** | Monophyletic genetic clade with high statistical support (≥80 for bootstrap for ML and ≥0.8 for posterior probability for Bayesian inference), with an intragenotype average p-distance ≤0.03 (or 3% divergence) for both subgroups and an intergenotype average p-distances ≥0.09 (9% divergence) for RSV-A and ≥0.05 (5% divergence) for RSV-B. |
| --- | --- |
| **Subgenotype** | Monophyletic genetic clade with high statistical support (≥80 for bootstrap for ML and ≥0.8 for posterior probability for Bayesian inference) which arises from dichotomies of within a given genotype. In addition, average p-distances among the subgenotypes must be <0.09 (9% divergence) for RSV-A and <0.05 (5% divergence) for RSV-B. |
| **Lineage** | Well-supported genetic clade whose ancestral node diverged from the subgenotype ancestral node by ranges of 0.015 of patristic distances, identified in a ML phylogenetic tree. |

**Supplementary table 3. Procedure to genotype newly sequenced strains**

| **1** | G-ectodomain sequence alignment including representative sequences of each of the defined genotypes/subgenotypes/lineajes should be obtained (reference alignments available as supplementary files). The G-ectodomain encompasses from 4986 to 5579 nt of the reference genome for RSV-A A2 strain (GenBank Acc. No. NC_038235.1) and from 5017 to 5562 nt of the reference B1 for RSV-B (GenBank Acc. No. NC_001781.1). |
| --- | --- |
| **2** | Phylogenetic analyses of such alignments including query and reference sequences should be performed using two different inference methods (Maximum likelihood and Bayesian inference). |
| **3** | High statistical support in the node of the clade containing the query sequence should be confirmed (≥80% bootstrap and ≥0.8 posterior probability values). |
| **4** | Agreement between phylogenetic trees obtained by the two inference methods applied should be confirmed. In case of no agreement, possible misaligned sequences/regions and/or incorrectly parameters set for the phylogenetic analyses should be checked. |
| **5** | Identification of amino acid haplotypes defined in table 3 should support the allocated genotype/subgenotype/lineage. |

**Supplementary table 4.** **How to define a new genotype/subgenotype or a lineage?**

| RSV Genotype | When the divergence of a previous defined genotype, measured as average intragenotype genetic distance (p-distance) surpasses the cut-off value (>0.03), the definition of a new genotype should be considered by dividing the original genotype in the minimum number of monophyletic genetic clades which meet the criteria of genotype definition.  When a new genetic clade is clustered outside all the defined genotypes, the estimation of the average intra/interclade p-distance should be estimated and if it meets the criteria of genotype definition, the new genotype should be assigned an appropriate name following the proposed ascending order nomenclature. |
| --- | --- |
| RSV subgenotype | Within a given genotype, when a new well-supported dichotomy of a genetic clade appears, and the average interclade p-distance meet the criteria of subgenotype definition, this new subgenotype should be named following the proposed nomenclature. |
| RSV lineage | When a genetic clade is suspected to be a new lineage, all the available sequences of that subgenotype should be analyzed. Starting with a ML phylogenetic tree rooted against a GA1/GB1 according to the RSV subgroup, and setting an increasing order of the nodes, the patristic distance should be measured starting with the zero-value set in the ancestral node of the corresponding subgenotype. If the ancestral node of a new suspected lineage falls beyond the cutoff value of 0.015 patristic distance from the previous lineage, this genetic clade should be considered as a new lineage and should be named following the proposed nomenclature. |

**Supplementary table 5.** Estimates of average genetic distances within GA1 and GB1 according different regions from the same alignment of complete genome sequences of RSV-A and B.

|  | Full-Genome | SH-G-F | SH | G | G-ectodomain | G 2^nd^ HR | F |
| --- | --- | --- | --- | --- | --- | --- | --- |
| RSV-A | 0.0079 SE:0.0005 (44) | 0.011 SE:0.001 (38) | 0.024 SE:0.005 (12) | 0.020 SE:0.002 (28) | 0.024 SE:0.002 (28) | 0.028 SE:0.004 (22) | 0.007 SE:0.001 (26) |
| RSV-B | 0.0076 SE:0.0004 (21) | 0.010 SE:0.001 (19) | 0.010 SE:0.005 (3) | 0.016 SE:0.002 (16) | 0.020 SE:0.002 (16) | 0.031 SE:0.005 (12) | 0.011 SE:0.001 (10) |

HR, hypervariable region.

Intra GA1/GB1 genetic distance calculated from the dataset of full genome sequences trimmed to obtain the different regions listed in the table and that were used to build the different trees of the supplementary figure 1 and 2. P- distances were calculated using MEGA7 software. Only non-identical sequences were considered for each region analyzed (detailed in parenthesis). Standard error estimates obtained by a bootstrap procedure (1000 replicates) are shown next to the average genetic distances. Value of p-distance intra GA1/GB1 for G ectodomain differ from the one calculated in the manuscript because this table only contains data from available RSV complete genomes.
