# Supplementary material for: Toward unified molecular surveillance of RSV: A proposal for genotype definition
Source: Influenza Other Respir Viruses. 2020 Feb 5;14(3):274–85. doi: 10.1111/irv.12715 (PMC7182609; doi:10.1111/irv.12715)
Supplement: Supplementary file 10 [file IRV-14-274-s010.docx]

**Supplementary figure 1.** Maximum likelihood tree of different regions of RSV-A and B genomes.

From an alignment of complete genome sequences per subgroup, different maximum likelihood trees were inferred considering different regions of the genome. Best nucleotide substitution model selection and trees were inferred with IQ-TREE software (The GTR+G was the most suitable model for most of the alignments, with exception of complete genome and the three surface glycoprotein alignments in which the GTR+I+G was selected for both subgroups. TIM+G was the model selected for both SH alignments) and 1000 replicates of ultrafast bootstrap approximation (UF bootstrap). Color scale represents UF bootstrap from 100 in green to 0 in red. Black dots at the end of the branches represent taxa.

**Supplementary figure 2.** Maximum clade credibility trees of different regions of RSV-A and B genomes.

From an alignment of complete genome sequences per subgroup, different trees were inferred considering different genes. Trees were inferred with BEAST software. Details of the parameters used are described in the Appendix. TreeAnnotator was used to summarize the information from a sample of trees in the maximum clade credibility tree once the convergence was reached. Color scale represents posterior probabilities from 0 in red to 1 in blue. Black dots at the end of the branches represent taxa.

**Supplementary figure 3. Comparison of the number and country representation between complete genomes and G ectodomain sequences.**

Pie charts of the country representation of the 1033 complete genomes (a) and 3764 G ectodomain (b) sequences available in GenBank up to February 2018. Countries are denoted in different colors and listed in descending order according to their abundance.

**Supplementary figure 4. Assessment of substitution saturation for different regions of the RSV-A and B genomes.**

Plot representation of the number of transitions and transversions versus the genetic distance with the most suitable model selected by IQ-TREE for different regions of the genome of RSV-A (a) and B (b) estimated with DAMBE software.

**Supplementary figure 5. Maximum likelihood tree of G ectodomain reference alignment for RSV-A.**

Maximum likelihood tree obtained with IQTREE using the proposed reference alignment for classification of RSV-A strain in described genotypes/subgenotypes/lineajes. Colors denotes genotype and subgenotype classification and parenthesis denotes lineages classification. Ultrafast bootstrap is shown in genotypes/subgenotypes/lineajes nodes. Signature amino acids of a given clade are also shown in nodes. Amino acids separated by ‘+’ symbol denote haplotype structure. In bold are highlight “main” amino acids (present only in the sequences of that clade), while italic highlighted “secondary” amino acids (present in more than one clade across the trees).

**Supplementary figure 6. Maximum likelihood tree of G ectodomain reference alignment for RSV-B.**

Maximum likelihood tree obtained with IQTREE using the proposed reference alignment for classification of RSV-B strain in described genotypes/subgenotypes/lineajes. Colors denotes genotype classification and parenthesis denotes lineages classification. Ultrafast bootstrap is shown in genotypes/subgenotypes/lineajes nodes. Signature amino acids of a given clade are also shown in nodes. Amino acids separated by ‘+’ symbol denote haplotype structure. In bold are highlight “main” amino acids (present only in the sequences of that clade), while italic highlighted “secondary” amino acids (present in more than one clade across the trees).
